# Operational characteristics of antiretroviral therapy clinics in Zambia: a time and motion analysis

**DOI:** 10.1186/s12913-019-4096-z

**Published:** 2019-04-24

**Authors:** Radhika P. Tampi, Taniya Tembo, Mpande Mukumba-Mwenechanya, Anjali Sharma, David W. Dowdy, Charles B. Holmes, Carolyn Bolton-Moore, Izukanji Sikazwe, Austin Tucker, Hojoon Sohn

**Affiliations:** 1 Department of Epidemiology, Johns Hopkins Bloomberg School of Public Health, 615 N. Wolfe Street, Baltimore, MD 21205 USA; 2 Centers for Infectious Disease Research (CIDRZ), Lusaka, Zambia

**Keywords:** Program efficiency, Time and motion studies, Antiretroviral therapy care evaluation, Antiretroviral therapy program monitoring, Allocation of resources, Workload

## Abstract

**Background:**

The mass scale-up of antiretroviral therapy (ART) in Zambia has taken place in the context of limited infrastructure and human resources resulting in many operational side-effects. In this study, we aimed to empirically measure current workload of ART clinic staff and patient wait times and service utilization.

**Methods:**

We conducted time and motion (TAM) studies from both the healthcare worker (HCW) and patient perspectives at 10 ART clinics throughout Zambia. Trained personnel recorded times for consecutive discrete activities based on direct observation of clinical and non-clinical activities performed by counselors, clinical officers, nurses, and pharmacy technicians. For patient TAM, we recruited consenting patients and recorded times of arrival and departure and major ART services utilized. Data from 10 clinics were pooled to evaluate median time per patient spent for each activity and patient duration of stay in the clinic.

**Results:**

The percentage of observed clinical time for direct patient interaction (median time per patient encounter) was 43.1% for ART counselors (4 min, interquartile range [IQR] 2–7), 46.1% for nurses (3 min, IQR 2–4), 57.2% for pharmacy technicians (2 min, IQR 1–2), and 78.5% for clinical officers (3 min, IQR 2–5). Patient workloads for HCWs were heaviest between 8 AM and 12 PM with few clinical activities observed after 2 PM. The length of patient visits was inversely associated with arrival time – patients arriving prior to 8 AM spent 61% longer at the clinic than those arriving after 8 AM (277 vs. 171 min). Overall, patients spent 219 min on average for non-clinical visits, and 244 min for clinical visits, but this difference was not significant in rural clinics. In comparison, total time patients spent directly with clinic staff were 9 and 12 min on average for non-clinical and clinical visits.

**Conclusion:**

Current Zambian ART clinic operations include substantial inefficiencies for both patients and HCWs, with workloads heavily concentrated in the first few hours of clinic opening, limiting HCW and patient interaction time. Use of a differentiated care model may help to redistribute workloads during operational hours and prevent backlogs of patients waiting for hours before clinic opening, which may substantially improve ART delivery in the Zambian context.

**Electronic supplementary material:**

The online version of this article (10.1186/s12913-019-4096-z) contains supplementary material, which is available to authorized users.

## Background

The World Health Organization’s (WHO) recommendation to expand antiretroviral therapy (ART) eligibility has led to a massive scale-up of ART worldwide, doubling the number of patients receiving ART services from 2010 to 2015 in eastern and southern Africa. However, existing clinical operations and management of ART services in these African countries have limited capacity to provide the services needed to match the scale-up of ART provision [1]. This has resulted in increasingly large workloads for clinical staff, long wait times for patients, and limited interaction between patients and clinicians. If not addressed, the resulting congestion, decreased service efficiency, and reduced patient retention may have long-term effects on the management of HIV treatment and transmission.

These issues are particularly salient in Zambia, where in 2014, 671,066 adults and children were receiving ART services from only 592 clinics, nearly all of which suffered from a critical human resource shortage [2, 3]. ART clinics in Zambia are generally integrated into higher-level general health facilities and ART staff members often have responsibilities outside of the ART clinic, which further exacerbates the shortage. These issues are expected to escalate as the Zambian Ministry of Health expanded the country’s ART eligibility guidelines in 2017 to include all HIV-positive individuals regardless of WHO clinical stage and CD4 count. Although several new ART clinics are being opened each year, understaffed clinics may face even greater challenges if this scale-up is not met with an increase in human resources and improved clinic efficiency. Several solutions have been put forth to alleviate the burden on the healthcare system and to reduce patient congestion and wait times. One proposed solution would be shifting healthcare delivery models from the “one-size-fits-all” framework of previous ART guidelines to differentiated models that are implemented based on the unique characteristics of the clinics and the patients they serve, such as reducing the number of clinical consultations for stable ART patients, creating patient-led counseling groups, and setting up ART refill sites outside the clinic.

Earlier studies have provided insight into how patient time spent post-triage is distributed among clinical stations in Zambia and have addressed potential solutions for ART clinic congestion, though in other country contexts [4–6]. However, there is little empirical evidence detailing the operational workflow of key clinical staff and the time distribution of patient visits in Zambian ART clinics. Such evidence could inform decisions about the relative impact of various interventions to reduce staff workload and patient wait times, ultimately improving clinic efficiency, quality of service and patient retention in long-term ART care.

We therefore conducted a time and motion (TAM) analysis to elucidate issues leading to clinic congestion from both the clinic staff and patient perspectives. This study aimed to empirically assess current operational characteristics of ART clinics, including the distribution of staff time among several key clinical activities, duration of patient-provider interactions, and variations in length for different types of patient visits.

## Methods

### Overview and rationale

The Center for Infectious Disease Research in Zambia (CIDRZ) is currently investigating the impact of differentiated care models for ART delivery in Zambia. These patient-centered approaches aim to better serve the needs of people living with HIV and “reduce unnecessary burdens on the health system”. [7] Differentiated care models could potentially increase the efficiency of clinics and patient retention for stable ART patients by decentralizing health services and making care more readily accessible. Before implementing these models, we sought to understand the current operational characteristics of ART clinics in Zambia and establish a baseline from which improvements and changes due to implementation of differentiated care models could be measured. The primary objectives for this analysis were to evaluate median time per patient spent by clinic staff in seven key activity groups, hourly workload distribution for clinic staff, patient time spent at the ART clinic for clinical versus non-clinical visits, and the relationship between the time patients arrive at the clinic and the length of their visit. We chose to report median time rather than average time as the distribution of time data for activities were skewed and had a wide range, thus we felt that using median time would be more representative of our data sample (see Fig. 2).

### Setting and site selection

TAM data collection was carried out at baseline, before the implementation of differentiated care models. We selected a subset of ten clinics in Zambia out of twenty-six clinics with CIDRZ-supported ART services, using purposive sampling to ensure diversity in geographical representation, clinic population, ART patient volume, and retention. We gathered baseline TAM data from clinic staff at all ten clinics and from patients at seven clinics (as logistical constraints limited our ability to obtain patient TAM data from three clinics) (Additional file 1: Table S1).

### Measurements

For clinic staff TAM, we developed a data collection form with standardized activity codes based on clinic site visits, interviews with clinic staff, and consultation with local experts. The TAM data collection team consisted of four to six trained personnel and one supervisor to record ART clinic staff activities in each clinic. The team held a sensitization meeting at each clinic prior to data collection to inform and assure clinic staff of both confidentiality and minimal interference with their daily activities, and obtained verbal consent for TAM data collection. The TAM data collection team arrived at the sites before opening (from 6:15–7:00) and left only after patient visiting hours ended (between 14:30–17:00) at the clinics. The team also collected staff rosters and scheduled TAM observation on a typical workday for the staff, excluding, for example, days on which staff saw only pediatric patients. On the day of the TAM, ART clinic staff members were given identification tags with specific codes to identify their roles and requested to indicate their names, roles, and codes on a roster. Each member of the data collection team was assigned to one or more stations (e.g. screening, triage, dispensary, and consultation room), depending on proximity of one station to another. Team members used a separate paper form for each staff member being observed to record consecutive activities and the corresponding start and end time for each code. Continuous activity was defined as an activity occurring without an interruption of more than one minute. Data collection for staff TAM occurred from February to April of 2016.

For patient TAM, our team recruited consecutive consenting ART patients and provided a form to record times of arrival and departure from the clinic and to circle all major ART services utilized (blood draw, adherence counseling, clinical consult, and pharmacy visit) during the visit. Upon completion of their ART clinic visit, patients were instructed to leave the forms at the pharmacy or with a study team member. We enrolled as many consenting patients as possible at each study site during the designated observation day. Patient TAM data collection occurred from September to October of 2016.

### Analysis

The TAM data were analyzed in two parts: 1) evaluation of the clinic staff time spent conducting ART clinic activities and 2) assessment of patient ART service utilization and total time spent at the clinic. Our outcomes of interest were the median time spent per patient for activities involving direct patient interaction and the proportion of patient time spent waiting to receive care during their clinic visit. We first pooled the data across the ten study clinics and grouped the 25 standardized activity codes into seven key activity groups as outlined in Table 1 for sub-group analyses. We used Stata (Version 14, StataCorp LLC, College Station, TX, USA) to analyze the TAM data.

**Table 1:** Distribution of Clinical Staff Time by Activity Group

Key Clinical Activity Groups	Counselors		Clinical Officers		Nurses		Pharmacy Tech	
	*n* = 53		*n* = 10		*n* = 31		*n* = 10	
	Time per Patient in minutes	Percent Daily Time	Time per Patient in minutes	Percent Daily Time	Time per Patient in minutes	Percent Daily Time	Time per Patient in minutes	Percent Daily Time
	(IQR)		(IQR)		(IQR)		(IQR)	
Triage	2 (2–4)	11%	–	–	3 (2–4)	31%	–	–
Counseling	4 (2–7)	32%	–	–	3 (1.5–4)	0.3%	–	–
Clinical Visit	6 (1–11)	0.2%	3 (2–5)	79%	5 (3–7)	14%	–	–
Pharmacy	3 (2–4)	3%	3.5 (2–5)	1%	1 (1–2)	1%	2 (1–2)	54%
Lab	–	–	–	–	4 (3–5)	9%	–	–
Administrative^a^	5.8	34%	0.1	2%	4	18%	3	23%
Other^a^	3.6	21%	0.9	19%	5	27%	3	23%

^a^To estimate per-patient time for non-patient specific activity groups (Administrative and Other), we divided total observed person-time spent by the estimated number of patients seen by the clinic staff. Administrative time spent per patient for nurses was calculated by dividing total observed time for administrative activities by the number of patients observed in triage

We focused our assessment on four categories of staff – adherence counselors, clinical officers, nurses, and pharmacy technicians. For each clinic staff category, we assessed the mean and median (with interquartile range) time spent on each activity group measured in minutes per patient. For activities not involving patient interaction (such as administrative work), we divided total observed person-time for the activity by the estimated number of patients seen by each staff category. The number of patients seen by each staff member was estimated by counting the encounters within the major activity group for each staff category – individual counseling sessions for adherence counselors, triage visits for nurses, clinical visits for clinical officers and pharmacy visits for pharmacy technicians. We also assessed temporal trends in workload with activity codes split into three categories: those involving direct patient interaction, administrative work, and other (including breaks, chatting, and waiting) for the four staff categories.

For patient TAM, we re-grouped service utilization into clinical or non-clinical visits, depending on whether a patient had seen a clinician (doctor/clinical officer/nurse prescriber) or not. Non-clinical visits were defined as those including only adherence counseling and pharmacy services, whereas clinical visits additionally included a clinical review, with or without laboratory services (generally a blood draw for CD4 count/viral load testing). Utilizing results from our staff TAM analysis, we estimated the median amount of time staff would spend on clinical versus non-clinical patient visits to assess total service time for patients based on the services they received. Total service time was then subtracted from total time spent at clinic to calculate median total wait time, both for patients arriving prior to clinic opening at 8 AM and patients arriving after 8 AM. Differences in length of clinical and non-clinical visits were calculated using the non-parametric Wilcoxon rank-sum test.

## Results

### Clinic staff TAM

**Fig. 1:**
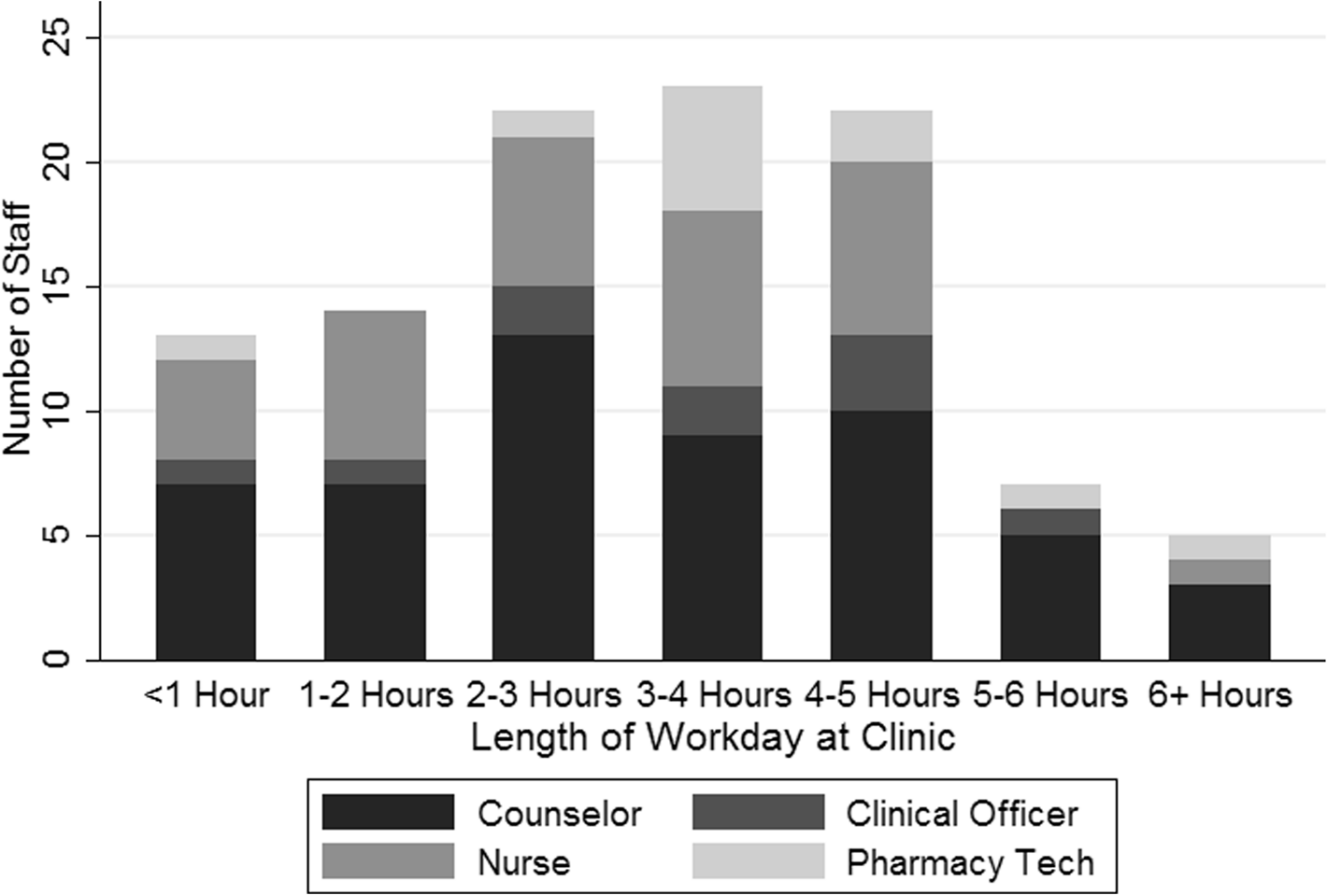
Observed Hours Worked at ART Clinic by Staff. Histogram of four key ART clinic staff showing frequency of observed hours worked at ART clinic**.** Out of 104 observed staff members, 92 were observed at the ART clinic for fewer than five hours on TAM day and 12 were observed at the clinic for 5+ hours

Across all 10 clinics, we observed 53 adherence counselors for a total of 7662 person-minutes, 10 clinical officers for 1412 person-minutes, 31 nurses for 4256 person-minutes, and 10 pharmacy technicians for 1932 person-minutes. On average, clinic staff members spent three to 4 hours working in the ART clinic per day. 27 of 53 counselors (51%) and 13 of 31 nurses (42%) were observed in the ART clinic for less than 3 hours. On average, counselors and clinical officers spent 3.2 h (SD: 1.7) at the clinic, versus 2.9 h (SD: 1.7) for nurses and 3.8 h (SD: 1.5) for pharmacy technicians (Fig. 1).

Direct patient interaction time for clinic personnel lasted a median of less than 5 minutes per encounter. Adherence counselors spent a median of 4 minutes (IQR: 2–7) with each patient for individual counseling, clinical officers spent 3 minutes (IQR: 2–5) on an individual patient consultation, nurses spent 3 minutes (IQR: 2–4) per patient at triage, and pharmacy technicians spent 2 minutes (IQR: 1–2) per patient for pharmacy visits (Table 1).

**Fig. 2:**
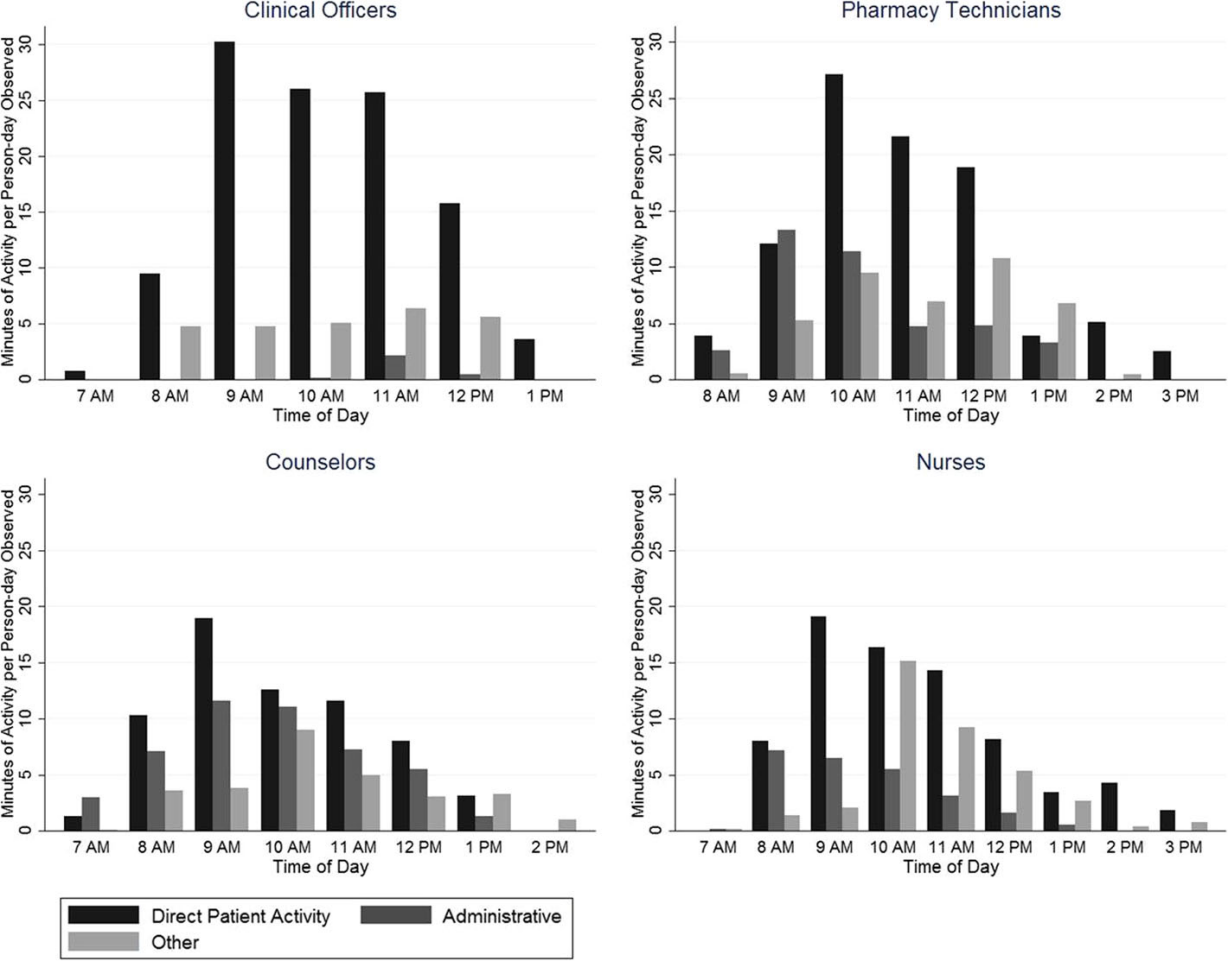
Clinic Staff Daily Time Distribution. Clinic staff activities were classified into three groups for the four staff categories. Direct patient interaction includes any activity involving one-to-one interaction between a patient and staff member, administrative work involves activities such as searching for files and updating patient registers, and activities such as chatting or taking a break were included under the “Other” category. Average time (in minutes) spent on each type of activity were graphed for each hour to show the distribution of activities throughout a work day. Times do not necessarily add up to 60 min as the numbers presented represent averages over all staff (some of whom did not contribute time in each time window presented). For all four categories, direct patient interaction is skewed right and peaks from 9 to 10 AM. There is no obvious trend for the administrative and other categories among the four staff categories

Assessment of the temporal distribution of clinical activities showed that, for all four staff categories, direct patient interaction peaks from 9 AM to 12 PM, with administrative and other activities following a similar, but less pronounced, trend (Fig. 2). Few activities were performed in any category of work after 1 PM.

Activities involving direct patient interaction (triage, adherence counseling, clinical visit, pharmacy visit and laboratory work) accounted for the majority (or near majority) of time spent for each type of clinic staff. Clinical officers spent 79% of observed person-time on activities involving direct patient interaction, while adherence counselors, nurses, and pharmacy technicians spent approximately 46, 55, and 57%, respectively. Of the four staff categories, counselors spent the most time on administrative activities at 34% of their observed person-time (Fig. 2).

### Patient TAM

From the patient perspective, we found that across all clinics, individual patients spent a median of 239 min (IQR: 171–300) per ART clinic visit. At rural clinics, median visit length was 253 min (IQR: 193–300) while at urban sites, median visit length was 232 min (IQR: 162–298). From our staff TAM analysis, we estimated that patients spent a median of 16 min (IQR: 12–19) receiving services for clinical visits and 10 min (IQR: 9–13) for non-clinical visits, with no significant difference between rural and urban clinics. For patients arriving prior to 8 AM (*n* = 230), median total time spent at clinic was 285 min (IQR: 234–332) for clinical visits and 275 min (232–328) for non-clinical visits, although this difference was not statistically significant. Median wait time prior to clinic opening was 80 min (IQR: 45–96) for clinical visits and 60 (IQR: 30–115) for non-clinical visits. For patients arriving after 8 AM at urban clinics, median total time spent at the clinic was 193 min (IQR: 137–254) for clinical visits, while the median for non-clinical visits was shorter by 65 min (128 min; IQR: 81–187; *p*-value = 0.000). Similarly, the difference in median total wait time between clinical and non-clinical visits for patients arriving after 8 AM at urban clinics was highly statistically significant (Table 2).

**Table 2:** Summary of Patient Time to Receive ART Services

	Median Time (Inter-Quartile Range)									
	Patients Arriving Before Clinic Opening (*n* = 230)						Patients Arriving After Clinic Opening (*n* = 166)			
Setting and Type of Visit	Total Time in Clinic	*P*-value	Total Time Waiting	*P*-value	Wait Time Prior to Clinic Opening	*P*-value	Total Time in Clinic	*P*-value	Total Wait Time	*P*-value
Rural Clinics										
Clinical	293 (259–310)	0.922	279 (244–291)	0.922	60 (40–120)	0.393	194 (128–217)	0.531	179 (109–202)	0.531
Non-clinical	288 (263–317)		277 (252–306)		90 (16–120)		206 (150–247)		195 (139–236)	
Urban Clinics										
Clinical	279 (222–335)	0.370	261 (203–318)	0.566	80 (51–90)	0.062	193 (137–254)	0.000	178 (122–235)	0.000
Non-clinical	262 (204–340)		250 (193–329)		60 (30–100)		128 (81–187)		117 (70–176)	
Overall										
Clinical	285 (234–332)	0.509	266 (217–312)	0.996	80 (45–96)	0.228	194 (134–242)	0.062	178 (117–227)	0.120
Non-clinical	275 (232–328)		264 (221–317)		60 (30–115)		153 (98–220)		142 (87–209)	

*p-value for difference between clinical and non-clinical visits, using the non-parametric Wilcoxon rank-sum test

**Fig. 3:**
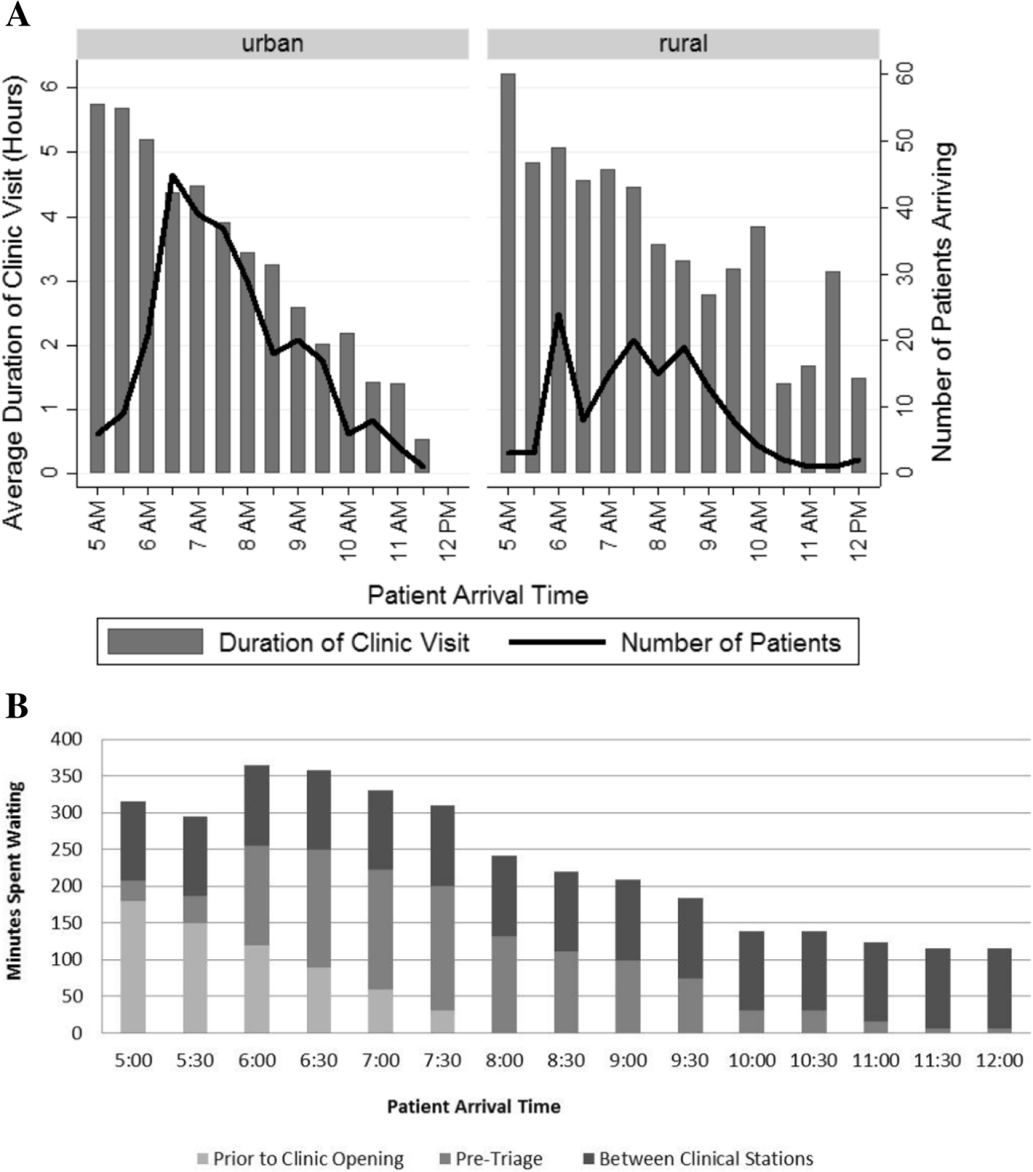
Patient Arrival and Congestion. **a** Bar graph showing a negative relationship between patient arrival time and the average duration of their clinic visit, superimposed with a line graph showing the number of patients arriving at the clinic within each half-hour block. Patient arrival peaks at 6:30 AM at urban clinics and 6 AM at rural clinics, with a sharp and steady decline in arrival after 7 AM in urban clinics and 8:30 AM in rural clinics. Patients arriving earlier in the day stayed at the clinic longer, on average, than patients arriving later in the day, creating a backlog of patients for clinical staff to see early in the morning. **b** Utilizing data from a recent CHAI study on patient wait times at Zambian ART clinics, we estimated how much of a patient’s time at the clinic is spent waiting versus receiving care. [6] Assuming that patients are seen on a first-come-first-serve basis, we found that patients arriving prior to clinic opening (before 8 AM) spend a majority of their time waiting for their files to be found and to be called into triage

Many patients arrived at the clinics hours before the clinic opened (*n* = 230; 58%). The earliest ART patient arrival recorded was at 5 AM, with clinics opening at 8 AM. No ART patients were observed arriving past 12 PM. Earlier patient arrival was inversely associated with the duration of the patient’s clinic visit; patients arriving at the clinic before 8 AM had a median visit length of 283 min (IQR: 234–330) while those arriving after 8 AM had a median visit length of 177 min (IQR: 115–231), a difference of 106 min (*p* < 0.0001) (Fig. 3).

## Discussion

This study highlights several important operational characteristics of ART clinics in Zambia that likely contribute to clinic congestion and distribution of workload for clinic staff. These inefficiencies may in turn result in lower quality of care and reduced patient retention. As differentiated care models of ART delivery are implemented in Zambia, understanding of these inefficiencies and developing solutions to mitigate these factors may become increasingly important.

First, our data show that the duration of each interaction between patients and clinic staff is limited, with most interactions lasting less than 4 minutes per patient, and with patients receiving services for only 16 min for a clinical visit when median total visit length at the clinic was over 4 hours. Similar findings were reported at HIV clinics in Kenya, reporting 4 minute counseling sessions at both urban and rural clinics. [5] In contrast, median time for clinical consultations for HIV patients in higher income countries last nearly five times longer, at around 16–20 min. [8] Length of patient-provider interaction has been shown to directly improve patient satisfaction, and patient satisfaction is associated with retention and adherence in HIV patients. [9–11] One potential cause of limited patient-provider interaction is patient frontloading due to early patient arrival, causing clinic staff to process high patient volumes between the hours of 8 AM to 12 PM. Our study data support anecdotes from other studies in Zambia and similar settings showing that patient arrival can occur as early as 4 AM. [4, 6] We also found that the earlier a patient arrives at the clinic, the longer they are likely to stay at the clinic. A similar study by the Clinton Health Access Initiative (CHAI) found that patients spend 2 hours on average visiting the clinic, but these results did not include patient time spent waiting prior to clinic opening and being called for triage. [6] Assuming that the distribution of time spent in each activity in our study is similar to those reported in this study, the potential drivers for patient wait time are likely to be waiting for the clinic to open (for patients arriving before or shortly after the clinic opens) and waiting to be called into triage. Later in the day, patients are likely processed faster, and total wait time is mostly comprised of rotating through clinical stations (Fig. 3). Differentiated care models could reduce the burden of patient frontloading by reducing the number of visits patients make to the clinic and thereby reducing the number of patients waiting prior to clinic opening.

Second, the average length of staff members’ workday is less than 4 hours at the ART clinic. Although our data collection methods did not capture activities conducted outside the ART clinic, short workdays at the ART clinic further limit the amount of time that staff can attend to patients. Not only are staff members spending a limited amount of their workday in the ART clinic, but a significant portion is spent not interacting directly with patients. During these observed hours spent in the ART clinic, staff spent from 20 to 53% of their time on average on administrative and other activities (i.e. waiting/chatting/taking breaks). Activities involving patient interaction taper off significantly around 1 PM, and the staff left at the ART clinic focus on administrative tasks until close of the clinic. Potential solutions to this include reducing the administrative workload of counselors and nurses by task shifting to registry clerks and data entry staff or implementing other interventions that allow patient interaction time to be spread across all operating hours for the clinic.

Ultimately, our study results should be interpreted with some care as the methodologies and the data have certain key limitations. First, TAM data collection was conducted only once per clinic, which limits this study to a cross-sectional analysis. Our data collection began after some patients had arrived at the clinic waiting room but before any clinic staff arrived. At some clinics, TAM data collection ended before the clinic had officially closed, but after all patient visits had concluded for the day. Following informal interviews with the clinic staff, we found that all activities occurring after 3 PM were administrative or preparing for the next day. Second, the short per-patient direct interaction times observed in our study are due to many operational and patient-level factors. However, we did not evaluate these factors (e.g. patient to staff ratio, motivation for patients to arrive at the clinic before opening, types of patient, etc.) in association with unit-time measures; therefore, it is difficult to ascertain which operational or patient-level factors have the greatest influence on current operational conditions in the Zambian ART clinics. Because our patient TAM data collection did not include time between each station that patients visited, we could not fully assess where and when bottlenecks in service provision occur and for which services patients wait the longest. Finally, generalizability may be limited, as our selected clinics may not be representative of all Zambian settings, nor of other ART delivery settings outside of Zambia – though we anticipate that some of the observed patterns hold in many other sub-Saharan African ART clinics.

## Conclusion

Our study empirically illustrates the current operational inefficiencies and limitations existing at Zambian public ART clinics, which result in highly concentrated workloads for HCWs and longer wait times for patients, particularly around clinic opening time. These findings point to the potential for improving overall patient care and reducing congestion at the ART clinics through the use of differentiated care models. If the majority of eligible stable ART patients can be enrolled in less intensive service delivery options, clinic staff may be able to redistribute their workload more evenly throughout the workday and focus on patients with acute medical needs. Furthermore, differentiated care models may obviate the perceived need of patients to arrive at the clinics very early and wait in long lines to receive relevant care. Ultimately, designing and implementing strategies to improve current ART clinic operational inefficiencies is imperative in improving patients’ ART care experience and the HCW work environment in Zambia.

## Additional file

Additional file 1: **Table S1.** Characteristics of Clinics Selected for TAM Study. Different characteristics of clinics selected for the TAM study, as a sub-set of the clinics testing the differentiated care model with CIDRZ. Characteristics include whether the clinic had the differentiated care intervention or control, whether it was located in a rural or urban area, the size of the clinic population, and the cumulative incidence of missed visits of the clinic population (used to check patient adherence to ART). (DOCX 26 kb)

## Abbreviations

ART: Antiretroviral Therapy
CD4: Cluster of Differentiation 4
CIDRZ: Centers for Infectious Disease Research Zambia
HCW: Healthcare Worker
HIV: Human Immunodeficiency Virus
IQR: Inter-Quartile Range
SD: Standard Deviation
TAM: Time and Motion
WHO: World Health Organization
