# Supplementary material for: Operational characteristics of antiretroviral therapy clinics in Zambia: a time and motion analysis
Source: BMC Health Serv Res. 2019 Apr 24;19:244. doi: 10.1186/s12913-019-4096-z (PMC6480736; doi:10.1186/s12913-019-4096-z)
Supplement: Supplementary file 1 — Table S1. Characteristics of Clinics Selected for TAM Study. Different characteristics of clinics selected for the TAM study, as a sub-set of the clinics testing the differentiated care model with CIDRZ. Characteristics include whether the clinic had the differentiated care intervention or control, whether it was located in a rural or urban area, the size of the clinic population, and the cumulative incidence of missed visits of the clinic population (used to check patient adherence to ART). (DOCX 26 kb) [file 12913_2019_4096_MOESM1_ESM.docx]

**Additional file 1**

| Name of Clinic | Intervention/  Control | Urban/  Rural | Clinic Population | Cumulative incidence of missed visit (> 14 days late) |
| --- | --- | --- | --- | --- |
| Community Adherence Group (CAG) | | | | |
| †Kalomo District Hospital | Intervention | Rural | 2890 | 0.36 |
| †Nsadzu | Intervention | Rural | 699 | 0.47 |
| Hofmeyr | Intervention | Rural | 328 | 0.54 |
| Nyimba District Hospital | Intervention | Rural | 3355 | 0.38 |
| Magoye | Intervention | Rural | 1522 | 0.54 |
| Lwiimba Rural HC | Control | Rural | 349 | 0.55 |
| †Mwase Lundazi | Control | Rural | 545 | 0.48 |
| †Mbaya Musuma | Control | Rural | 1014 | 0.32 |
| Sinda Rural HC | Control | Rural | 1694 | 0.40 |
| Pemba Main HC | Control | Rural | 1672 | 0.51 |
| Urban Adherence Group (UAG) | | | | |
| †Kabwata | Intervention | Urban | 3757 | 0.50 |
| †Kalingalinga | Intervention | Urban | 7502 | 0.40 |
| Petauke District Hospital | Intervention | Urban | 7181 | 0.39 |
| George | Intervention | Urban | 8241 | 0.54 |
| Manungu | Intervention | Urban | 1583 | 0.49 |
| Nakambala Clinic | Control | Urban | 2171 | 0.50 |
| Matero Reference | Control | Urban | 10599 | 0.57 |
| †Nang’ongwe Clinic | Control | Urban | 3067 | 0.48 |
| Chipata General Hospital | Control | Urban | 7961 | 0.40 |
| †Chelstone | Control | Urban | 8402 | 0.43 |
| Fast Track (FT) | | | | |
| †Makeni | Intervention | Urban | 4720 | 0.35 |
| †Matero Main | Intervention | Urban | 5048 | 0.46 |
| Mtendere | Control | Urban | 4054 | 0.42 |
| Bauleni | Control | Urban | 3284 | 0.51 |
| Chazanga | Control | Urban | 3042 | 0.57 |
| Ngombe | Control | Urban | 4190 | 0.65 |

**Table S1**: Characteristics of clinics selected for TAM study

†Sites chosen for TAM study
